# CWMS-GAN: A small-sample bearing fault diagnosis method based on continuous wavelet transform and multi-size kernel attention mechanism

**DOI:** 10.1371/journal.pone.0319202

**Published:** 2025-04-11

**Authors:** Shun Yu, Zi Li, Jialin Gu, Runpu Wang, Xiaoyu Liu, Lin Li, Fusen Guo, Yuheng Ren

**Affiliations:** 1 School of Systems and Computing, University of New South Wales, Canberra, Australia; 2 Guanghua School of Management, Peking university, Beijing, China; 3 Faculty of Science and Engineering, Southern Cross University, Gold Coast, Australia; 4 School of Business Economics, European Union University, Montreux, Switzerland; Ataturk University, TÜRKIYE

## Abstract

In industrial production, obtaining sufficient bearing fault signals is often extremely difficult, leading to a significant degradation in the performance of traditional deep learning-based fault diagnosis models. Many recent studies have shown that data augmentation using generative adversarial networks (GAN) can effectively alleviate this problem. However, the quality of generated samples is closely related to the performance of fault diagnosis models. For this reason, this paper proposes a new GAN-based small-sample bearing fault diagnosis method. Specifically, this study proposes a continuous wavelet convolution strategy (CWCL) instead of the traditional convolution operation in GAN, which can additionally capture the signal’s frequency domain features. Meanwhile, this study designed a new multi-size kernel attention mechanism (MSKAM), which can extract the features of bearing vibration signals from different scales and adaptively select the features that are more important for the generation task to improve the accuracy and authenticity of the generated signals. In addition, the structural similarity index (SSIM) is adopted to quantitatively evaluate the quality of the generated signal by calculating the similarity between the generated signal and the real signal in both the time and frequency domains. Finally, we conducted extensive experiments on the CWRU and MFPT datasets and made a comprehensive comparison with existing small-sample bearing fault diagnosis methods, which verified the effectiveness of the proposed approach.

## Introduction

Rolling bearings, known as the “joints of industry,” are critical components in rotating machinery and are widely used in various fields of industrial production [1]. However, due to prolonged high-speed operation, complex system environments, improper assembly, and inadequate lubrication, rolling bearings often experience failures during operation, which can affect the normal functioning of the equipment [2]. Once the equipment fails, it may cause economic loss, or it may cause safety accidents and threaten people’s lives [3,4]. Therefore, accurately diagnosing the failure state of rolling bearings is crucial. This not only helps reduce maintenance costs and improve production efficiency but also enables timely elimination of safety hazards, thereby safeguarding people’s lives and property.

With the continuous improvement of computer computing power and artificial intelligence technology, various intelligent bearing fault diagnosis models [5] have emerged in response to the ensuing challenges of massive data processing and the demand for real-time monitoring. The existing intelligent rolling bearing fault diagnosis methods can be categorized into two main categories: machine learning-based [6–8] and deep learning-based [9–12]. Moosavian et al. [6] proposed a fault diagnosis method for the main radial bearings of internal combustion engines based on power spectral density and K-Nearest Neighbors (KNN). Li et al. [7] analyzed multiple features reflecting bearing conditions and selected 12 critical features as inputs to Artificial Neural Network (ANN) for fault recognition. Zheng et al. [8] extracted multi-scale fuzzy entropy from bearing fault signals and used it as a feature input to Support Vector Machine (SVM) for fault classification. Deng et al. [9] introduced the Deep Boltzmann Machine (DBM) model to identify the fault conditions of rolling bearings, extracting time-domain, frequency-domain, and time-frequency domain features as input parameters for the DBM model, finding that combining time-domain and time-frequency features of the signal is more suitable for DBM in achieving bearing fault recognition. Fuan et al. [10] developed an Adaptive Deep Convolutional Neural Network (CNN) for rolling bearing fault diagnosis, utilizing Particle Swarm Optimization to determine the main parameters of the deep CNN model. Xia et al. [11] proposed an intelligent fault diagnosis method based on the stacked denoising autoencoder (AE) deep neural network, which applies the denoising autoencoder to unlabeled data to extract representative features. Ding et al. [12] introduced a method for extracting fault features of transmission devices using Adaptive Variational Mode Decomposition (AVMD) and employed a Deep Belief Network (DBN) for pattern recognition.

Although the methods mentioned above have achieved excellent results in fault diagnosis, they rely on a large amount of data for model training. However, in practical engineering, monitoring data for mechanical equipment often suffers from low usability and low-value density, resulting in a severe scarcity of bearing fault data [13]. Therefore, fault diagnosis of bearings under small-sample conditions has become a research focus in this field [14]. To address this challenge, researchers have proposed various solutions, among which data sampling is one of the standard processing techniques. This method expands the data quantity of minority classes by undersampling the abundant samples and oversampling the scarce ones, thereby alleviating the problem of sample scarcity [15–17]. However, data sampling merely increases the quantity of the original data without fundamentally enhancing the diversity of the data, resulting in limited improvement effects from this approach.

Meta-learning accumulates learning experience by training on multiple tasks to enhance a model’s ability to adapt quickly to new tasks, effectively addressing the small-sample problem. Models based on meta-learning [18,19] typically employ task partitioning and rapid adaptation strategies to improve the effectiveness of small-sample bearing fault diagnosis. In contrast, transfer learning guides target domain learning by leveraging information obtained from the source domain to address the issue of insufficient training data in the target domain. It often uses strategies such as model pre-training and fine-tuning to enhance the effectiveness of small-sample bearing fault diagnosis [20,21]. While meta-learning and transfer learning offer practical solutions for small-sample fault diagnosis, neither method has thoroughly resolved the issue of limited data in fault diagnosis.

In recent years, more researchers have focused on using Generative Adversarial Networks (GAN) and their variants [22–25] to address the challenge of insufficient bearing data in practical engineering applications. Liang et al. [26] extracted time-frequency image features from one-dimensional signals using wavelet transformation and employed GAN to generate additional time-frequency image samples to compensate for the lack of actual data. Yang et al. [27] proposed CGAN-2D-CNN, which converts vibration signals into two-dimensional grayscale images and utilizes Conditional Generative Adversarial Networks (CGAN) to generate grayscale images that assist actual samples in fault diagnosis. However, when converting signals from one dimension to two-dimensional images, inherent vibration features from the original one-dimensional bearing signals, such as periodicity and fault pulse characteristics, may be lost, adversely affecting the model’s diagnostic performance. Li et al. [28] introduced an Auxiliary Class Wasserstein GAN with Gradient Penalty (ACWGAN-GP) that directly generates one-dimensional bearing signals to alleviate the problem of data imbalance in fault diagnosis. Yang et al. [29] presented a Structure Similarity-based Generative Adversarial Network (SSGAN), which, combined with an improved MobileNetv3, is used for small-sample bearing fault diagnosis. Luo et al. [30] proposed a Conditional Deep Convolutional GAN (CDCGAN) for imbalanced fault diagnosis, conducting a detailed study on its effectiveness. Wang et al. [31] proposed E-GAN, which utilizes a Deep Convolutional GAN (DCGAN) to generate more samples and integrates a K-means clustering algorithm for small-sample fault classification. Gao et al. [32] combined transformers with convolutional neural networks to propose ICoT-GAN, which comprehensively extracts global and local temporal features from the original signals, thereby generating high-quality signals. Chen et al. [33] developed FMRGAN, which addresses the checkerboard artefacts caused by transposed convolutions in the generator, aiming to produce high-quality fault data to enhance the training dataset and improve the performance of fault diagnosis models under limited data conditions.

Despite existing small-sample bearing fault diagnosis methods based on Generative Adversarial Networks (GAN) having achieved significant results, they typically focus on either time-domain or frequency-domain features when generating signals and lack a systematic analysis of the reliability of the generated signals. This may lead to issues with the effectiveness and accuracy of the generated data in practical applications. Therefore, this paper proposes a novel small-sample bearing fault diagnosis method based on GANs and conducts an in-depth analysis of the reliability of the generated samples to ensure that the generated data can more effectively assist in enhancing the performance of fault diagnosis models. The contributions of this paper are as follows:

1. This paper proposes a new GAN-based small-sample bearing fault diagnosis method. A large number of experiments are carried out on CWRU and MFPT data sets, which fully proves the method’s feasibility and effectiveness.
2. This study proposes a continuous wavelet convolution strategy (CWCL) to reconfigure the conventional convolutional layers of the generator and discriminator in GAN. The aim is to enable the model to capture detailed information about the signal in both time and frequency domains and thus generate higher-quality samples.
3. This paper designs a multi-size kernel attention mechanism (MSKAM) and applies it to GAN. MSKAM can extract feature information from bearing vibration signals at different scales and adaptively select the most important features for the generation task, thereby improving the accuracy and authenticity of the generated signals.

## Relevant theory

### Deep Convolutional Generative Adversarial Network

**Fig 1:**
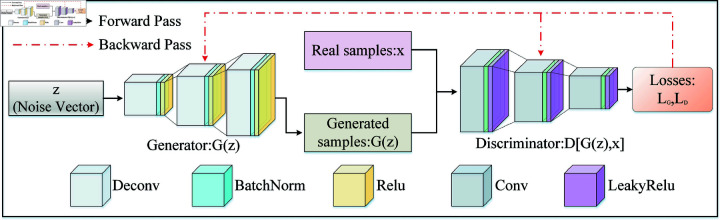
The detailed structure of Deep Convolutional Generative Adversarial Network (DCGAN).

It is well known that Deep Convolutional Generative Adversarial Network (DCGAN) is identical to GAN [22] in principle; the main difference is that DCGAN replaces the traditional fully-connected layer in GAN with a convolutional layer, which enhances the model’s ability to capture sample features. As shown in Fig 1, DCGAN [24] consists of the generator and the discriminator, and its core is the adversarial training process between the generator and the discriminator. In this process, the goal of the discriminator is to maximize the ability to distinguish actual data from generated data, and its loss function is usually expressed as follows:


$$\begin{array}{ccc} L_{D}=E_{x∼p_{data}(x)}[\text{log}D(x)]+E_{z∼p(z)}[\text{log}(1-D(G(z)))] & & \end{array}$$ (1)

where *G*(*z*) denotes the generated data by the generator based on the random noise *z*, while *D*(*x*) and *D*(*G*(*z*)) denote the discriminator’s output probabilities of the actual data *x* and the generated data *G*(*z*), the discriminator tries to maximize $L_{D}$, i.e., to keep *D*(*x*) as close to 1 as possible (i.e., to identify the actual data correctly), while keeping *D*(*G*(*z*)) as close to 0 as possible (i.e., to identify the generated data as forged correctly). The generator’s goal is to produce realistic data that the discriminator cannot distinguish between true and false. Therefore, the loss function of the generator is as follows:


$$\begin{array}{ccc} L_{G}=E_{z∼p(z)}[\text{log}(1-D(G(z)))] & & \end{array}$$ (2)

The generator tries to minimize the loss $L_{G}$ (i.e., make *D*(*G*(*z*)) close to 1), aiming to make the discriminator mistakenly believe that the generated data is actual. During the model training process, the generator and the discriminator continuously optimize their respective performances by confronting each other until both sides reach a Nash equilibrium, i.e., the generated data by the generator is indistinguishable from the actual data, and the discriminator is no longer able to judge the authenticity accurately.

### Continuous Wavelet Transform

Due to the influence of load changes, rotational speed fluctuations, and changes in the state of the bearing itself, rolling bearing signals often show prominent non-stationary characteristics [34]. In recent years, wavelet theory has opened up a new path in fault diagnosis [35], owing to its unique advantage in dealing with non-stationary signals. Continuous Wavelet Transform (CWT) [36], one of the commonly used time-frequency analysis tools, is based on the core idea of convolving the signal with a mother wavelet function to extract the time-frequency features of the signal at different scales and time intervals. The mathematical expression of CWT is as follows:


$$\begin{array}{ccc} W(a,b)=\frac{1}{\sqrt{\left|a\right|}}\int_{-\infty }^{\infty }x(t)\psi^{*}\left(\frac{t-b}{a}\right)dt & & \end{array}$$ (3)

In Eq (3), *x*(*t*) represents the input signal, and *τ* .  is the mother wavelet function. By adjusting the scale factor *a*, the wavelet can be compressed or stretched to capture components of different frequencies. In contrast, the translation factor *b* controls the movement of the wavelet function along the time axis, allowing the analysis of signal features at different time positions. The normalization factor $1∕\sqrt{\left|a\right|}$ ensures energy consistency across different scales. In short, CWT provides both time and frequency information of the signal and performs multi-scale analysis through the dilation and translation of the wavelet function, making it widely used for time-frequency feature extraction of non-stationary signals.

### Convolutional Block Attention Module

Traditional convolutional neural networks [37–39] still have some limitations in feature expressiveness, so Woo et al. [40] proposed the Convolutional Block Attention Module (CBAM), which effectively enhances the network’s attention to essential features while suppressing the influence of unimportant features, thus improving the model performance.

**Fig 2:**
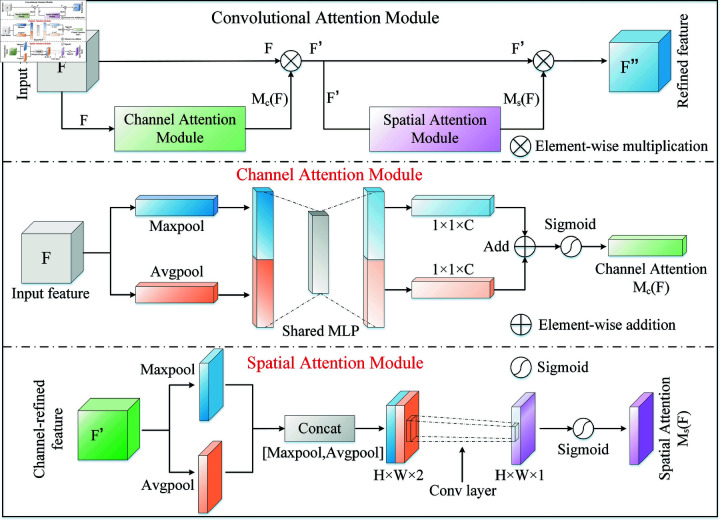
The detailed structure of Convolutional Block Attention Module (CBAM).


As shown in Fig 2, CBAM consists of two separate sub-modules: the channel and spatial attention modules. The role of the channel attention module is to weight each channel of the feature map to highlight key features. Specifically, given an input feature $F\in {\mathbb{R}}^{H\times W\times C}$, where *H*, *W*, and *C* represent the height, width, and number of channels of *F*, respectively. *F* is first compressed into two *i* = 1 , . . . , *N* feature vectors by average pooling and max pooling and processed through a shared fully connected layer (MLP). Next, the output features of the MLP are summed channel-by-channel and activated by sigmoid to generate the final channel attention feature $M_{c}(F)\in {\mathbb{R}}^{C}$. Finally, $M_{c}(F)$ is subjected to an element-by-element multiplication operation with the input feature map *F*, and the result $F^{\prime }$ is then fed into the spatial attention module.

$$\begin{array}{cc} M_{c}(F) & =\sigma (MLP(AvgPool(F))+MLP(MaxPool(F))) \end{array}$$ (4)

$$\begin{array}{cc} F^{\prime } & =M_{c}(F)⊗F \end{array}$$ (5)

The spatial attention module first compresses the feature map $F^{\prime }$ across the channel dimension, generating two feature maps of size *S* ( *t* ) . , obtained through average pooling and max pooling operations. These two feature maps are then concatenated along the channel dimension to form a feature map of size *θ* ( *τ* ) , which is convolved using a 7×7 convolution layer. After passing through a sigmoid activation function, the spatial attention weight $M_{s}(F)\in {\mathbb{R}}^{H\times W}$ is generated. Finally, $M_{s}(F)$ is multiplied element-wise with $F^{\prime }$ to obtain the final weighted feature map $F^{\prime \prime }$. The formula is as follows:


$$\begin{array}{ccc} M_{s}(F) & =\sigma \left(f{\;}^{7\times 7}\left(\left[AvgPool(F^{\prime });MaxPool(F^{\prime })\right]\right)\right) & \end{array}$$ (6)

$$\begin{array}{ccc} {}[6pt]F^{\prime \prime } & =M_{s}(F)⊗F^{\prime } & \end{array}$$ (7)

In Eq (6), $f{\;}^{7\times 7}$ represents a convolution operation with a kernel size 7×7. $[AvgPool(F^{\prime });$ $MaxPool(F^{\prime })]$ refers to the concatenation of $AvgPool(F^{\prime })$ and $MaxPool(F^{\prime })$ along the channel dimension. In Eq (7), $F^{\prime \prime }$ represents the key features that are more helpful to the current task after filtering irrelevant redundant information, thus improving the inherent feature representation ability of the model.

## Proposed methods

**Fig 3:**
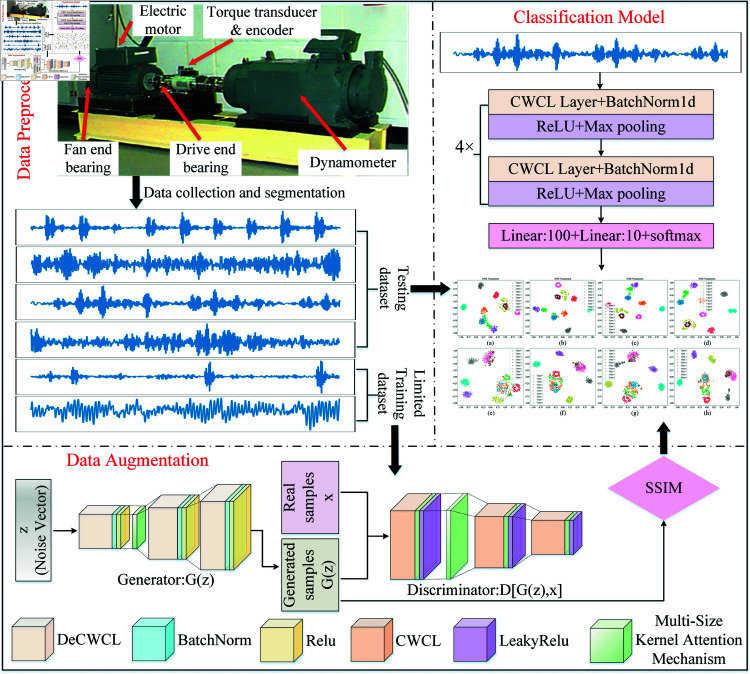
The overall framework of small sample fault diagnosis method based on CWMS-GAN.

Fig 3 illustrates the overall framework of the proposed small-sample rolling bearing fault diagnosis method, which consists of three parts: data preprocessing, data augmentation, and a classification model.

First, the data preprocessing module collects bearing signals and divides them into limited training and test sets. Next, the data augmentation module optimizes the original DCGAN using Continuous Wavelet Convolution (CWCL) and Multi-Size Kernel Attention Mechanism (MSKAM) and trains on the limited training set to generate new samples. Subsequently, the generated signals are evaluated for quality using SSIM. Finally, the limited training set is mixed with the generated signals and input into the classification model for training, thereby achieving the final fault classification.

### Continuous Wavelet Convolutional Layer

Although conventional convolution performs well in many bearing fault diagnosis tasks, it cannot effectively capture transient variations and bursty signal features because it uses a fixed-size convolution kernel to extract features. In addition, conventional convolution cannot provide both time and frequency information, making a comprehensive understanding of the spectral and temporal characteristics of the signal difficult. The conventional convolution operation is defined as follows:


$$\begin{array}{ccc} y(t)=(x*w)(t)=\int_{-\infty }^{\infty }x(\tau )w(t-\tau )\;d\tau & & \end{array}$$ (8)

where *w*(*t*) is a convolution kernel of fixed size, and *x*(*t*) and *y*(*t*) denote the input and output signals, respectively. As described in the subsection “Continuous Wavelet Transform,” the CWT can provide signal time and frequency information and perform multi-scale analysis by expanding and translating the wavelet function to extract the signal features effectively. However, in practical applications, the selection of the mother wavelet is crucial, and the Morlet wavelet [41] combines sinusoidal and Gaussian functions, which has good time and frequency localization ability, and its expression is


$$\begin{array}{ccc} \psi (t)=e^{j2\pi f_{0}t}e^{-\frac{t^{2}}{2\sigma^{2}}} & & \end{array}$$ (9)

In Eq (9), $e^{-\frac{t^{2}}{2\sigma^{2}}}$ represents a Gaussian function, $e^{j2\pi f_{0}t}$ represents a sinusoidal wave with frequency $f_{0}$, $f_{0}$ denotes the center frequency of the sinusoidal wave, which is set to 5, and *j* ≠ *i* .  represents the standard deviation of the Gaussian function, with its value set to $\frac{1}{2\pi f_{0}}$. Therefore, this paper selects the Morlet wavelet as the mother wavelet function and applies it to the CWT to construct a Continuous Wavelet Convolutional Layer (CWCL). Subsequently, the CWCL will replace the traditional convolutional layers in the GAN and fault diagnosis classifier, as shown in the following formula:


$$\begin{array}{ccc} & y(a,b)=\int_{-\infty }^{\infty }x(t)\psi_{a,b}(t)\;dt & \end{array}$$ (10)

$$\begin{array}{ccc} \left[6pt\right] & \psi_{a,b}(t)=\frac{1}{\sqrt{\left|a\right|}}e^{j2\pi f_{0}\frac{\left(t-b\right)}{a}}e^{-\frac{{\left(t-b\right)}^{2}}{2a^{2}\sigma^{2}}} & \end{array}$$ (11)

Compared with traditional convolutional operations, CWCL enables GAN to extract time-domain features and capture signal frequency variations. This results in a more comprehensive understanding of the time-frequency characteristics of non-smooth signals, which helps to improve the quality of the signals generated by the GAN.

### Multi-size kernel attention mechanism

**Fig 4:**
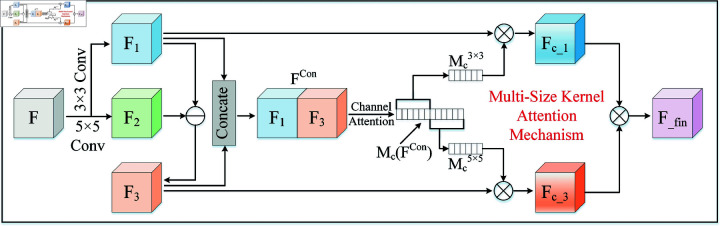
The structure of multi-size kernel attention mechanism.

As mentioned earlier, the fault signals of bearings usually have non-stationary characteristics, i.e., their frequency components change continuously over time. The channel attention module of traditional CBAM only uses a single-scale convolution kernel, which makes it difficult to capture the variable frequency features in the fault signal effectively. Therefore, this paper improves the channel attention module in CBAM and proposes a new multi-size kernel attention mechanism (MSKAM), as shown in Fig 4, which is capable of extracting features under multi-frequency components through convolution kernels of different scales to enhance the model’s adaptability to non-smooth fault signals.

In this study, we first use two different sizes of convolution kernels, 3×3 and 5×5, to perform convolution operations on the input feature maps *F*, respectively, so as to obtain the feature maps $F_{1}$ and $F_{2}$. Since feature maps $F_{1}$ and $F_{2}$ of different scales contain a large amount of redundancy information, we removed this redundancy information before performing channel concatenation. The formula is as follows:


$$\begin{array}{ccc} & F_{1}=F_{Conv}^{3\times 3};F_{2}=F_{\text{Conv}}^{5\times 5};F_{3}=F_{1}-F_{2} & \end{array}$$ (12)

$$\begin{array}{ccc} \left[6pt\right] & F^{Con}=Concatenate[F_{1};F_{3}] & \end{array}$$ (13)

where $F_{3}$ denotes the feature map after removing the coexisting redundant information in $F_{1}$ and $F_{2}$, while $F_{Conv}$ denotes the feature map obtained by channel concatenation the feature maps $F_{1}$ and $F_{3}$ and then calculating its channel attention weight $M_{c}(F^{Con})$. Next, $M_{c}(F^{Con})$ is divided into two parts: $M_{c}^{3\times 3}$ and $M_{c}^{5\times 5}$. Finally, the channel attention weights of the branches are fused to obtain the final channel attention feature map $F_{fin}$. The whole process can be represented as


$$\begin{array}{ccc} & F_{c\text{\_}1}=M_{c}^{3\times 3}⊗F_{1};F_{c\text{\_}3}=M_{c}^{5\times 5}⊗F_{3} & \end{array}$$ (14)

$$\begin{array}{ccc} \left[6pt\right] & F_{fin}=F_{c\text{\_}1}⊗F_{c\text{\_}3} & \end{array}$$ (15)

Here, $M_{c}^{3\times 3}$ and $M_{c}^{5\times 5}$ represent the channel attention weights corresponding to $F_{1}$ and $F_{3}$, respectively. $F_{c\text{\_}1}$ is the enhanced feature map obtained by element-wise multiplication of $M_{c}^{3\times 3}$ and $F_{1}$, while $F_{c\text{\_}3}$ is the enhanced feature map obtained by element-wise multiplication of $M_{c}^{5\times 5}$ and $F_{3}$.

### Time and frequency feature similarity

The CWMS-GAN proposed in this paper improves the original DCGAN by enabling the model to capture both time-domain features and frequency variations of the signals to generate higher-quality samples to solve the problem of limited bearing fault data in practice. Wang et al. proposed the Structural Similarity Index (SSIM) [42], which measures the similarity between two images by simulating the perceptual characteristics of the human visual system. More importantly, as mentioned in their paper, SSIM is not limited to image processing. In fact, as a symmetric similarity measure, SSIM can also be used to assess the similarity between any two signals. Therefore, in this paper, SSIM is used to compute the similarity between the generated signal and the real signal in both time and frequency domains for quantitative evaluation of the generated signal.

Suppose now there are a real signal *r* and a generated signal *g* and $r,g\in {\mathbb{R}}^{1\times 1024}$; the steps of SSIM are described as follows:

- Calculate the mean as well as the variance of *r* and *g*, respectively, with the mathematical formulas shown in Eqs (16) and (17).
  $$\begin{array}{ccc} & \mu_{r}=\frac{1}{N}\overset{N}{\underset{i=1}{\sum }}r_{i},\mu_{g}=\frac{1}{N}\overset{N}{\underset{i=1}{\sum }}g_{i} & \end{array}$$ (16)
  $$\begin{array}{ccc} \left[6pt\right] & \sigma_{r}^{2}=\frac{1}{N-1}\overset{N}{\underset{i=1}{\sum }}{\left(r_{i}-\mu_{r}\right)}^{2},\sigma_{g}^{2}=\frac{1}{N-1}\overset{N}{\underset{i=1}{\sum }}{\left(g_{i}-\mu_{g}\right)}^{2} & \end{array}$$ (17)
  where $\mu_{r}$ and $\mu_{g}$ denote the mean of *r* and *g*, respectively, and $\sigma_{r}^{2}$ and $\sigma_{g}^{2}$ indicate the variance of *r* and *g*. *N* is the length of the signal, and $r_{i}$ and $g_{i}$ represent the value of the *i*-th point of *r* and *g*, respectively.
- Calculate the covariance $\sigma_{rg}$ between *r* and *g* as shown in Eq (18).
  $$\begin{array}{ccc} \sigma_{rg}=\frac{1}{N-1}\overset{N}{\underset{i=1}{\sum }}(r_{i}-\mu_{r})(g_{i}-\mu_{g}) & & \end{array}$$ (18)
- Define three comparison functions for luminance comparison, contrast comparison, and structure comparison, as shown in Eq (19).
  $$\begin{array}{ccc} l(r,g)=\frac{2\mu_{r}\mu_{g}+C_{1}}{\mu_{r}^{2}+\mu_{g}^{2}+C_{1}},c(r,g)=\frac{2\sigma_{r}\sigma_{g}+C_{2}}{\sigma_{r}^{r}+\sigma_{g}^{2}+C_{2}},s(r,g)=\frac{\sigma_{rg}+C_{3}}{\sigma_{r}\sigma_{g}+C_{3}} & & \end{array}$$ (19)
  where $C_{1}={\left(K_{1}\times L\right)}^{2}$, $C_{2}={\left(K_{2}\times L\right)}^{2}$, $C_{3}=\frac{C_{2}}{2}$. *L* is the maximum magnitude of the signal *r*, and the values of $K_{1}$ and $K_{2}$ are 0.01 and 0.03, respectively. Where $K_{1}$ and $K_{2}$ are chosen based on the recommendations of Wang et al. in their work [42].
- Finally, the three comparison functions are multiplied to obtain the final SSIM index, as shown in Eq (20).
  $$\begin{array}{ccc} SSIM(r,g)=\frac{\left(2\mu_{r}\mu_{g}+C_{1}\right)\left(2\sigma_{rg}+C_{2}\right)}{\left(\mu_{r}^{2}+\mu_{g}^{2}+C_{1}\right)\left(\sigma_{r}^{2}+\sigma_{g}^{2}+C_{2}\right)} & & \end{array}$$ (20)

The SSIM values range from –1 to 1, where a value close to 1 indicates high similarity between the two signals, and a value close to -1 indicates complete dissimilarity. For the bearing signals used in this study, which are one-dimensional with a consistent sampling length of 1024 points, we compute the SSIM directly on the entire signal rather than using a sliding window approach. This decision is based on the following considerations:(1) the sampling length of the signal is relatively short, and the distribution of characteristics is relatively uniform; (2) bearing vibration signals are typically periodic, where overall trends or frequency characteristics are more critical than minor local variations; (3) avoiding the use of a sliding window reduces computational complexity. Consequently, the SSIM is calculated for the entire signal, effectively treating the sliding window size as *g* ( ⋅ ) .

In this way, the similarity between signals *r* and *g* is first assessed based on their time-domain characteristics. Subsequently, their frequency-domain representations are obtained by applying the Fast Fourier Transform (FFT) to *r* and *g*, and the SSIM calculation is repeated on their respective absolute values rather than the complex-valued outputs of the FFT. It is important to note that the SSIM values computed for the time and frequency domains are reported and analyzed separately without being combined into a single similarity metric.

## Experiments and results analysis

In this paper, extensive experiments are conducted on two publicly available rolling bearing datasets, CWRU and MFPT, to evaluate the performance of the proposed CWMS-GAN. First, we use limited training samples to train CWMS-GAN to generate new samples. Then, these generated samples are fused with the original data to expand the training dataset, and the diagnostic performance of CWMS-GAN is evaluated on the test set. Finally, by comparing with multiple state-of-the-art methods, the effectiveness and superiority of the proposed method are further validated. Additionally, we use the SSIM metric to quantitatively assess the quality of the generated samples by CWMS-GAN.

The method is implemented using the PyTorch framework and Python 3.8. The development environment is Pycharm 2022, and the experimental equipment is equipped with an Intel Core i7-12700H processor (16 GB RAM) and an RTX 3080 graphics card.

### Dataset description

The CWRU dataset [43], collected by Case Western Reserve University, includes equipment such as a motor, torque sensor, dynamometer, and electronic controller. Single-point defects were introduced onto bearings using electric spark erosion to simulate fault conditions. Vibration signals were then collected under four load conditions (0, 1, 2, and 3 hp) at a sampling frequency of 12 kHz. The data covers four categories: healthy (H), inner race fault (IF), outer race fault (OF), and rolling element fault (BF). Each category is further subdivided into three damage sizes: 0.007, 0.014, and 0.021 inches.

The MFPT dataset [44], provided by the American Society for Mechanical Failure Prevention Technology, contains multiple categories of data collected at different loads and sampling frequencies, organized into 23 categories. Specifically, the following categories are included: (1) three normal data and three outer ring fault data, all with a load of 270 lbs, an input shaft speed of 25 Hz, and a sampling frequency of 97,656 Hz. (2) seven sets of outer ring fault data, with an input shaft speed of 25 Hz, a sampling frequency of 48,828 Hz, and loads of 25, 50, 100, 150, 200, 250, and 300 lbs. (3) seven sets of inner ring fault data with the same rotational speed of 25 Hz, sampling frequency of 48828 Hz, and loads of 0, 50, 100, 150, 200, 250, and 300 lbs. Details of the CWRU and MFPT datasets used in the experiments in this study are shown in Table 1.

**Table 1:** Detailed information about the CWRU and MFPT rolling bearing datasets.

Dataset	Fault location	Labels	Load	Diameter/ Sample frequency	Sampling points	Training set sample size	Test set sample size
CWRU	Roller fault	Class 0	0 hp	0.007 inch	1024	5/10/20/40	100
		Class 1	0 hp	0.014 inch	1024	5/10/20/40	100
		Class 2	0 hp	0.021 inch	1024	5/10/20/40	100
	Inner race	Class 3	0 hp	0.007 inch	1024	5/10/20/40	100
		Class 4	0 hp	0.014 inch	1024	5/10/20/40	100
		Class 5	0 hp	0.021 inch	1024	5/10/20/40	100
	Outer race	Class 6	0 hp	0.007 inch	1024	5/10/20/40	100
		Class 7	0 hp	0.014 inch	1024	5/10/20/40	100
		Class 8	0 hp	0.021 inch	1024	5/10/20/40	100
	Normal	Class 9	0 hp	-	1024	5/10/20/40	100
MFPT	Inner race	Class 0	0 lbs	48828 Hz	1024	5/10/20/40	100
		Class 1	50 lbs	48828 Hz	1024	5/10/20/40	100
		Class 2	150 lbs	48828 Hz	1024	5/10/20/40	100
		Class 3	300 lbs	48828 Hz	1024	5/10/20/40	100
	Outer race	Class 4	0 lbs	48828 Hz	1024	5/10/20/40	100
		Class 5	50 lbs	48828 Hz	1024	5/10/20/40	100
		Class 6	150 lbs	48828 Hz	1024	5/10/20/40	100
		Class 7	270 lbs	97656 Hz	1024	5/10/20/40	100
		Class 8	300 lbs	48828 Hz	1024	5/10/20/40	100
	Normal	Class 9	270 lbs	97656 Hz	1024	5/10/20/40	100

### Fault diagnosis under limited data condition

In this section, we first analyze the performance of the CWMS-GAN-based bearing fault diagnosis method under different training sample sizes when the number of training samples is limited (i.e., no additional generated samples are used). To simulate the limited number of bearing fault samples in real working conditions, we selected 5, 10, 20, and 40 samples from each category to construct the training dataset. The test dataset was fixed at 100 samples per category. To ensure the robustness of the results, we conducted 10 repetitions of the experiment, each with a different random split of the data into training and test sets, allowing us to obtain the average accuracy across these repetitions. As shown in Table 2, as the number of training samples increased from 5 to 40 per class, the accuracy of the CWRU dataset increased from 76.54*i* = 1 , . . . , *N* .  to 96.67*g* ( ⋅ ) , and the accuracy of the MFPT dataset increased from 71.25*a* = 0 to 93.37*g* ( ⋅ ) . These results indicate that as the number of training samples increases, the model is able to capture more feature information from the original data, which significantly enhances the generalization ability of the classification model and improves the fault diagnosis accuracy. Therefore, the number of training samples is crucial for deep learning-based fault diagnosis models, which also highlights the necessity of conducting bearing fault diagnosis research under small sample conditions.

**Table 2:** The diagnostic average accuracy on the test sets after attaching different numbers of generated samples to the CWRU and MFPT training sets.

dataset	Number of training samples for each class	vAccuracy of the original dataset	Accuracy of the augmented datasets			
			10 additional generated samples per class	20 additional generated samples per class	30 additional generated samples per class	40 additional generated samples per class
CWRU	5	76.54%	85.62%	91.25%	93.04%	94.31%
	10	84.27%	91.04%	93.69%	95.23%	96.46%
	20	91.79%	94.55%	96.35%	97.47%	98.79%
	40	96.67%	97.88%	98.92%	99.43%	99.83%
MFPT	5	71.25%	82.56%	88.31%	90.57%	91.74%
	10	81.86%	88.42%	90.76%	92.44%	94.35%
	20	89.06%	91.55%	93.24%	94.85%	96.09%
	40	93.37%	95.18%	96.82%	97.33%	97.94%

This section also explores the impact of the number of generated samples on the diagnostic performance of the model. Specifically, in the case of training CWMS-GAN to generate new fault samples by selecting 5, 10, 20, and 40 samples for each class, respectively, 10, 20, 30, and 40 samples are generated sequentially to expand the original dataset and assist the classifier for training. The results are shown in Table 2. It can be easily found that the increase in the number of generated samples significantly improves the diagnostic accuracy of the model regardless of the number of original training samples. Taking the CWRU dataset as an example, the diagnostic accuracy of the model is only 76.54*i* = 1 , . . . , *N* when the number of original training samples is only 5, and with the increase in the number of generated samples from 10 to 40, the accuracy gradually increases from 85.62*a* = 0 to 94.31*a* = 0. On the MFPT dataset, similarly, the diagnostic accuracy is only 71.25*P* ( *A* = 1 ) = 0 . 5 when the original training sample is 5, but the accuracy improves to 91.74*P* ( *C* = 1 ) = 0 . 5 when the generated samples are increased to 40. These results show that even under the condition of limited training samples, CWMS-GAN is able to generate sufficiently high-quality samples to significantly make up for the lack of raw data and effectively improve the performance of the diagnostic model.

**Fig 5:**
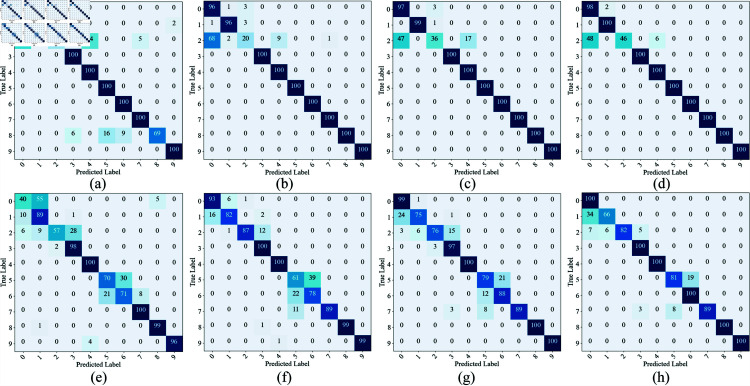
Confusion matrix of CWMS-GAN on the test datasets of CWRU and MFPT.(a)-(d):CWRU 5,10,20,40;(e)-(h):MFPT 5,10,20,40.

In order to visualize the effectiveness of CWMS-GAN in recognizing different fault types on the CWRU and MFPT datasets, in this section, the confusion matrix is used to visualize the classification results of CWMS-GAN with the number of training samples of 5, 10, 20, and 40, respectively, and supplemented with the generation of an additional 40 samples, as shown in Fig 5. The diagonal part of the confusion matrix represents the number of correctly classified samples, while the off-diagonal part shows the number of misclassified samples. It can be clearly seen that the recognition effect of CWMS-GAN on different fault types gradually improves as the number of training samples increases. The confusion matrix presented here is based on the median results of 10 repetitions for each experimental group. With fewer samples (e.g., 5 samples per class), there may be some confusion between certain fault types, resulting in a small number of misclassifications. However, as the number of training samples increases to 10, 20, and 40, the percentage of correct classifications improves significantly, and misclassifications in the non-diagonal part of the class gradually decrease. This trend is reflected in each fault type in both the CWRU and MFPT datasets, especially in the categories where the fault patterns are closer, which suggests that the samples generated by the CWMS-GAN significantly help the model to learn the subtle features, especially when the number of samples is limited, which effectively enhances the model’s fault differentiation ability.

**Fig 6:**
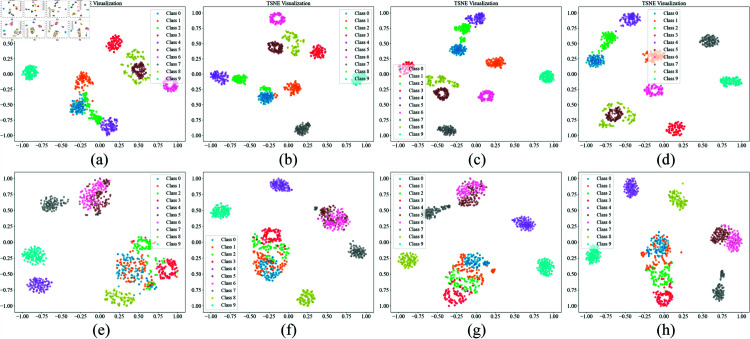
Distribution of t-SNE features of CWMS-GAN on the test datasets of CWRU and MFPT. (a)-(d):CWRU 5,10,20,40; (e)-(h):MFPT 5,10,20,40. Each category has 100 points, representing the number of samples per category in the test dataset.

To further evaluate the feature extraction capability of CWMS-GAN, this paper also examines the distribution of feature vectors in the embedding space. Specifically, under conditions where each class has 5, 10, 20, and 40 samples, along with an additional 40 generated samples, t-distributed Stochastic Neighbor Embedding (t-SNE) is used to reduce the high-dimensional features extracted from the last convolutional layer of the classifier into two-dimensional space for visualization (as shown in Fig 6). The results demonstrate that, on both the CWRU and MFPT datasets, the features extracted by CWMS-GAN exhibit distinct inter-class separation and high intra-class compactness in the two-dimensional space, highlighting the model’s ability to distinguish between different fault categories as well as the consistency within each category. Similarly, the t-SNE plots are generated based on the median results obtained from 10 repetitions of each experimental group.

### Comparison with the state-of-the-art methods

In this section, the CWMS-GAN method proposed in this paper is compared with several current mainstream small-sample bearing fault diagnosis methods, including DCGAN, CGAN-2D-CNN, ACWGAN-GP, SSGAN, CDCGAN, E-GAN, and FMRGAN, to further validate its advantages. The comparison is conducted on the CWRU dataset. The choice of the CWRU dataset for this comparison is based on its widespread use in the literature, which allows for a more direct and meaningful comparison with the majority of existing studies. Additionally, most research in this field has utilized the CWRU dataset along with private datasets to validate model performance, while the MFPT dataset has been less commonly used. Therefore, to ensure a consistent and fair comparison with other reported results, we have focused on the CWRU dataset in this study.

The comparison results are shown in Table 3. It should be noted that these comparison methods are all based on the data provided in their original papers. As seen from Table 3, when the number of training samples per class is 5 and 20, the diagnostic accuracy of CWMS-GAN is significantly higher than most of the comparison methods and only slightly lower than FMRGAN. Furthermore, compared to the training time of about 45 minutes for the CGAN-2D-CNN model, the training time of the proposed CWMS-GAN is about 52 minutes under the condition that only 10 samples per class are used as training data, and an additional 40 samples are generated. After training, the actual inference time of the CWMS-GAN in the bearing fault diagnosis task is about 5 minutes. Overall, the experimental results show that CWMS-GAN performs excellently under the condition of small samples and can generate high-quality fault samples and improve the accuracy of diagnostic models effectively, showing its advantages in the task of small-sample bearing fault diagnosis.

**Table 3:** Comparative experimental results under the CWRU dataset

Method	Training Samples	Addition generated samples	Diagnostic average accuracy
DCGAN [24] (2015)	5	20	82.55%
ACWGAN-GP [28] (2020)	5	20	80.85%
CDCGAN [30] (2021)	5	20	80.9%
CGAN-2D-CNN [27] (2021)	12	48	96.33%
E-GAN [31] (2021)	20	180	98.62%
SSGAN [29] (2022)	5	20	84.6%
FMRGAN [33] (2024)	5	20	94.77%
	15	40	99.02%
CWMS-GAN	5	20	91.25%
	10	40	96.46%
	20	40	98.79%
	40	40	99.83%

### Ablation experiment

This section conducted detailed ablation experiments to assess the specific impact of the two components, CWCL and MSKAM, on the model’s classification accuracy. The related results are shown in Table 4. The experiments were conducted under different sample size conditions, specifically with each category having 5, 10, 20, and 40 samples, as well as an additional 40 generated samples. Consistent with the previous experimental setup, each set of experiments is repeated 10 times to ensure the robustness of the results. From Table 4, it can be observed that CWCL improved the performance by 2.25*i* = 1 , . . . , *N*, 1.62*λ*, 0.75*ν*, and 1.38 ( 0 , *s* )  under different sample sizes, while MSKAM showed a more significant improvement, with increases of 3.07*k* = 1 , . . . , *K*, 2.1*b* = 2, 1.49*i* ∈ { 1 , 2 , ⋯ , *n* } , and 1.58*%*. This indicates that both CWCL and MSKAM can effectively enhance the model’s classification performance, with MSKAM demonstrating a more pronounced advantage. When combining CWCL, MSKAM, and TFPS, the classification accuracy of the model reached its optimal level, fully showcasing the synergistic effect among the three components.

**Table 4:** Results of ablation experiments on model classification accuracy.

CWCL	MSKAM	Diagnostic average accuracy of different samples			
		5	10	20	40
✗	✗	89.31%	92.46%	95.79%	97.45%
✓	✗	91.56%	94.08%	96.54%	98.83%
✗	✓	92.38%	94.56%	97.28%	99.03%
✓	✓	94.31%	96.46%	98.79%	99.83%

### Evaluation of generated samples

The proposed CWMS-GAN employs a class-by-class approach to generating samples rather than training to generate all classes of data simultaneously. Notably, the above process is run in parallel. For each category, each sample is selected sequentially from the real samples, and an SSIM calculation is performed with all the generated samples for that category. Finally, the quality of the generated samples for that category is evaluated using the average of all calculations.

In this subsection, we select five samples from each category of the CWRU dataset to constitute the training set and train DCGAN, DCGAN+CWCL, DCGAN+MSKAM, and our proposed CWMS-GAN model to generate an additional 40 samples. To evaluate the quality of the generated samples, we use the SSIM metrics in the time and frequency domains, respectively, and the results are shown in Table 5.

**Table 5:** The SSIM evaluation values of generated signals and real signals on CWRU dataset

Method		Fault location			Average
DCGAN	time domain	Class 0	Class 1	Class 2	Class 3	Class 4	Class 5	Class 6	Class 7	Class 8	Class 9	0.758
		0.6975	0.7360	0.6460	0.7990	0.7894	0.8072	0.8224	0.8086	0.6528	0.8214	
	frequency domain	Class 0	Class 1	Class 2	Class 3	Class 4	Class 5	Class 6	Class 7	Class 8	Class 9	0.731
		0.7062	0.6834	0.6480	0.7336	0.7572	0.7584	0.8171	0.7674	0.6277	0.8100	
DCGAN +CWCL	time domain	Class 0	Class 1	Class 2	Class 3	Class 4	Class 5	Class 6	Class 7	Class 8	Class 9	0.766
		0.7026	0.7207	0.6834	0.8008	0.8103	0.8107	0.8326	0.8003	0.6638	0.8314	
	frequency domain	Class 0	Class 1	Class 2	Class 3	Class 4	Class 5	Class 6	Class 7	Class 8	Class 9	0.745
		0.7090	0.7014	0.6765	0.7551	0.7539	0.7765	0.8342	0.7551	0.6590	0.8322	
DCGAN +MSKAM	time domain	Class 0	Class 1	Class 2	Class 3	Class 4	Class 5	Class 6	Class 7	Class 8	Class 9	0.785
		0.7584	0.7361	0.7043	0.7924	0.8231	0.8307	0.8475	0.7909	0.7035	0.8649	
	frequency domain	Class 0	Class 1	Class 2	Class 3	Class 4	Class 5	Class 6	Class 7	Class 8	Class 9	0.77
		0.7215	0.7380	0.6966	0.7855	0.8065	0.7980	0.8458	0.7517	0.6997	0.8519	
CWMS-GAN (DCGAN +CWCL +MSKAM)	time domain	Class 0	Class 1	Class 2	Class 3	Class 4	Class 5	Class 6	Class 7	Class 8	Class 9	0.818
		0.7787	0.7949	0.7304	0.8261	0.8377	0.8583	0.9084	0.8159	0.7349	0.8983	
	frequency domain	Class 0	Class 1	Class 2	Class 3	Class 4	Class 5	Class 6	Class 7	Class 8	Class 9	0.795
		0.7537	0.7740	0.7058	0.8104	0.8242	0.8054	0.8735	0.7966	0.7251	0.8729	

Observing the results, firstly, the generated samples of all models are generally more similar to the real samples in the time domain. This phenomenon can be explained by the fact that since the models directly take the time-domain signals as inputs during the training process, the models naturally retain the time-domain features and laws when learning to generate the samples, which makes the generated samples highly similar to the real samples in the time domain. Secondly, we note that the four models’ similarity in Class 2 and Class 8 categories is lower in both time and frequency domains. We speculate this may be related to the fault characteristics or signaling patterns specific to Classes 2 and 8. The data in these categories have more complex features in both the time and frequency domains, and the models may have some limitations in capturing these complex features, which affects the quality of the generated samples. In addition, through comparative analysis, we find that MSKAM contributes more to improving the quality of model-generated samples than CWCL, which is consistent with our analysis in the ablation experiments. Finally, by comparing the average SSIM values of the four model-generated samples with the real samples in the time and frequency domains, it can be found that the samples generated by CWMS-GAN at different fault locations in the CWRU dataset are the most similar to the real signals, which further proves the effectiveness of the proposed method in fault diagnosis tasks.

## Conclusion

In this paper, a bearing fault diagnosis method based on CWMS-GAN is proposed, aiming to complement the real samples by generating high-quality signals to cope with the problem of limited rolling bearing data under real working conditions that lead to the degradation of the performance of traditional deep-learning-based diagnostic models. A continuous wavelet convolution (CWCL) and a Multi-Size Kernel Attention Mechanism (MSKAM) are designed in CWMS-GAN to improve the traditional DCGAN so that it can capture both time and frequency domain information of the signal while extracting features from vibration signals at different scales and adaptively selecting key features to improve the accuracy and authenticity of the generated signal. In addition, the SSIM evaluation metric is adopted to quantify the quality of the generated data by calculating the similarity between the generated signal and the real signal in terms of time and frequency domain features.

The experimental results on the CWRU dataset show that compared to other small-sample diagnostic methods, CWMS-GAN exhibits higher classification accuracies for the number of training samples of 5 and 20, proving its excellent diagnostic capability under limited data conditions. The visualization analysis of the confusion matrix and t-SNE feature distribution further shows that CWMS-GAN has significant differentiation between different categories, while it exhibits good consistency among similar samples, verifying its effective feature extraction capability. In addition, it is not difficult to find that the SSIM values of the signals generated by CWMS-GAN and the real signals in the time domain and the frequency domain are relatively satisfactory, indicating that CWMS-GAN can generate high-quality bearing fault signals.

Although CWMS-GAN performs well in small-sample fault diagnosis, it relies on fully labeled data, which is often complicated and costly to label under real-world working conditions. Future research can combine semi-supervised learning methods so that the model can still effectively extract fault features under limited labeling conditions, thus improving its adaptability and diagnostic capability in real scenarios.

## References

[1] Jiang H, Li C, Li H. An improved EEMD with multiwavelet packet for rotating machinery multi-fault diagnosis. Mech Syst Signal Process. 2013;36(2):225–39.

[2] Xu F, Ding N, Li N et al. A review of bearing failure modes, mechanisms and causes. Eng Fail Anal. 2023:107518.

[3] Grebe M, Molter J, Schwack F. Damage mechanisms in pivoting rolling bearings and their differentiation and simulation. Bearing World J. 2018;3:71–86.

[4] Liu R, Yang B, Zio E. Artificial intelligence for fault diagnosis of rotating machinery: a review. Mech Syst Signal Process. 2018;108:33–47.

[5] Hakim M, Omran AAB, Ahmed AN. A systematic review of rolling bearing fault diagnoses based on deep learning and transfer learning: Taxonomy, overview, application, open challenges, weaknesses and recommendations. Ain Shams Eng J. 2023:14(4);101945.

[6] Moosavian A, Ahmadi H, Tabatabaeefar A. Comparison of two classifiers; K-nearest neighbor and artificial neural network, for fault diagnosis on a main engine journal-bearing. Shock Vib. 2013;20(2):263–72.

[7] Li C, De Oliveira JV, Cerrada M. Observer-biased bearing condition monitoring: from fault detection to multi-fault classification. Eng Appl Artif Intell. 2016;50:287–301.

[8] Zheng J, Pan H, Cheng J. Rolling bearing fault detection and diagnosis based on composite multiscale fuzzy entropy and ensemble support vector machines. Mech Syst Signal Process 2017;85:746–59.

[9] Deng S, Cheng Z, Li C. Rolling bearing fault diagnosis based on Deep Boltzmann machines. In: 2016 Prognostics and System Health Management Conference (PHM-Chengdu). IEEE; 2016, pp. 1–6.

[10] Fuan W, Hongkai J, Haidong S. An adaptive deep convolutional neural network for rolling bearing fault diagnosis. Meas Sci Technol. 2017:28(9);095005.

[11] Xia M, Li T, Liu L. Intelligent fault diagnosis approach with unsupervised feature learning by stacked denoising autoencoder. IET Sci Meas Technol. 2017;11(6):687–95.

[12] Ding F, Xia Y, Tian J. An AVMD method based on energy ratio and deep belief network for fault identification of automation transmission device. IEEE Access. 2021;9:150088–97.

[13] Gao T, Yang J, Jiang S. A novel fault detection model based on vector quantization sparse autoencoder for nonlinear complex systems. IEEE Trans Industr Inform. 2022;19(3):2693–704.

[14] Dixit S, Verma NK. Intelligent condition-based monitoring of rotary machines with few samples. IEEE Sensors J. 2020;20(23):14337–46.

[15] Li X, Zhang W, Ding Q. Intelligent rotating machinery fault diagnosis based on deep learning using data augmentation. J Intell Manuf. 2020;31(2):433–52.

[16] Qian M, Li YF. A weakly supervised learning-based oversampling framework for class-imbalanced fault diagnosis. IEEE Trans Reliab. 2022;71(1):429–42.

[17] Zhu QX, Wang XW, Zhang N. Novel K-Medoids based SMOTE integrated with locality preserving projections for fault diagnosis. IEEE Trans Instrum Meas. 2022;71:1–8.

[18] Chen J, Hu W, Cao D. A meta-learning method for electric machine bearing fault diagnosis under varying working conditions with limited data. IEEE Trans Industr Inform. 2022;19(3):2552–64.

[19] Su H, Xiang L, Hu A. A novel method based on meta-learning for bearing fault diagnosis with small sample learning under different working conditions. Mech Syst Signal Process. 2022;169:108765.

[20] Mao W, Liu Y, Ding L. A new structured domain adversarial neural network for transfer fault diagnosis of rolling bearings under different working conditions. IEEE Trans Instrum Meas. 2020;70:1–13.33776080

[21] Wang Z, He X, Yang B. Subdomain adaptation transfer learning network for fault diagnosis of roller bearings. IEEE Trans Industr Electron. 2021;69(8):8430–9.

[22] Goodfellow I, Pouget-Abadie J, Mirza M. Generative adversarial nets. Adv Neural Inf Process Syst. 2014;27.

[23] Mirza M, Osindero S. Conditional generative adversarial nets. arXiv. 2014. arXiv:1411.1784.

[24] Radford A. Unsupervised representation learning with deep convolutional generative adversarial networks. arXiv. 2015. arXiv:1511.06434.

[25] Odena A, Olah C, Shlens J. Conditional image synthesis with auxiliary classifier GANs. In: International Conference on Machine Learning. PMLR; 2017, pp. 2642–51.

[26] Liang P, Deng C, Wu J. Intelligent fault diagnosis of rotating machinery via wavelet transform, generative adversarial nets and convolutional neural network. Measurement. 2020;159:107768.

[27] Yang J, Liu J, Xie J. Conditional GAN and 2-D CNN for bearing fault diagnosis with small samples. IEEE Trans Instrum Meas. 2021;70:1–12.33776080

[28] Li Z, Zheng T, Wang Y. A novel method for imbalanced fault diagnosis of rotating machinery based on generative adversarial networks. IEEE Trans Instrum Meas. 2020;70:1–17.33776080

[29] Yang X, Liu B, Xiang L. A novel intelligent fault diagnosis method of rolling bearings with small samples. Measurement. 2022;203:111899.

[30] Luo J, Huang J, Li H. A case study of conditional deep convolutional generative adversarial networks in machine fault diagnosis. J Intell Manuf. 2021;32(2):407–25.

[31] Wang R, Zhang S, Chen Z. Enhanced generative adversarial network for extremely imbalanced fault diagnosis of rotating machine. Measurement. 2021;180:109467.

[32] Gao H, Zhang X, Gao X. ICoT-GAN: integrated convolutional transformer GAN for rolling bearings fault diagnosis under limited data condition. IEEE Trans Instrum Meas. 2023.

[33] Chen Y, Qiang Y, Chen J. FMRGAN: feature mapping reconstruction GAN for rolling bearings fault diagnosis under limited data condition. IEEE Sensors J. 2024.

[34] Chen R, Liu J, Tang J. Vibration characteristics analysis of rolling bearing rotor system considering radial clearance and outer raceway defect. Adv Mech Eng. 2023:15(4);16878132231167670.

[35] Yan R, Gao RX, Chen X. Wavelets for fault diagnosis of rotary machines: a review with applications. Signal Process. 2014;96:1–15.

[36] Kumar HS, Upadhyaya G. Fault diagnosis of rolling element bearing using continuous wavelet transform and K-nearest neighbour. Mater Today Proc. 2023;92:56–60.

[37] He K, Zhang X, Ren S. Deep residual learning for image recognition. In: Proceedings of the IEEE Conference on Computer Vision and Pattern Recognition. 2016, pp. 770–8.

[38] Huang G, Liu Z, Van Der Maaten L. Densely connected convolutional networks. In: Proceedings of the IEEE Conference on Computer Vision and Pattern Recognition. 2017, pp. 4700–8.

[39] Howard AG. Mobilenets: Efficient convolutional neural networks for mobile vision applications. arXiv. 2017. arXiv:1704.04861.

[40] Woo S, Park J, Lee JY. CBAM: convolutional block attention module. In: Proceedings of the European Conference on Computer Vision (ECCV). 2018, pp. 3–19.

[41] Shao H, Xia M, Wan J. Modified stacked autoencoder using adaptive Morlet wavelet for intelligent fault diagnosis of rotating machinery. IEEE/ASME Trans Mechatron. 2021;27(1):24–33.

[42] Wang Z, Bovik AC, Sheikh HR. Image quality assessment: from error visibility to structural similarity. IEEE Trans Image Process. 2004;13(4):600–12. 15376593 10.1109/tip.2003.819861

[43] Smith WA, Randall RB. Rolling element bearing diagnostics using the Case Western Reserve University data: a benchmark study. Mech Syst Signal Process. 2015;64:100–31.

[44] Dataset M. Society for Machinery Failure Prevention Technology. 2020.

